# *“I always wondered if my baby is able to feel my love for them” - * Development and pilot testing of two behavioural feedback strategies designed to improve maternal self-efficacy

**DOI:** 10.12688/wellcomeopenres.23564.2

**Published:** 2025-05-09

**Authors:** Alessandra Prioreschi, Deborah M James, Rebecca M Pearson, Antonia Smith, Shane A Norris, Kirsten L Rennie

**Affiliations:** 1 SAMRC Developmental Pathways for Health Research Unit, University of the Witwatersrand, School of Clinical Medicine, Department of Pediatrics, Johannesburg, Gauteng, South Africa; 2 Education and Social Research Institute, Manchester Metropolitan University Faculty of Health and Education, Manchester, England, UK; 3 MRC Epidemiology Unit, University of Cambridge School of Clinical Medicine, Cambridge, England, UK

**Keywords:** Early childhood development, personalised, parenting intervention, behaviour change, sensitivity

## Abstract

**Background:**

The aim of this pilot study was to describe the development of, and test the acceptability and feasibility of providing feedback on two behaviours in the context of supportive approaches to improve maternal self-efficacy. We hypothesised that providing individual behavioural feedback to mothers in a socially vulnerable context, when later embedded in supportive intervention approaches, may improve maternal self-efficacy and ultimately promote infant development.

**Methods:**

Feedback was developed following expert consultation with working groups, and included graphical feedback on mother and infant movement behaviours measured using accelerometry, as well as video feedback of mother-infant interactions using first person observation head cameras. Mothers wore the devices for one week, following which individual feedback on movement behaviours and mother-infant interactions was delivered at the Chris Hani Baragwanath Academic Hospital. We adapted an established and published strengths based approach as a guide for health workers to feedback video and accelerometer data. Feasibility and acceptability were tested by conducting focus group discussions with a sample of 8 mothers of infants aged 4-months from South Africa using a semi-structured interview guide.

**Results:**

Mothers included in this study were generally single, unemployed, and less than half had completed formal schooling. Most mothers had two or more children, and lived in informal housing (such as shacks and small, temporary prefabricated structures) with only outdoor toilet facilities available. The feedback was found to be both feasible, and largely acceptable in this context and compliance was 100%.

**Conclusion:**

We have been able to develop feedback with the potential to sensitise mothers to their infants’ behaviours. When embedded in a supportive intervention, these feedback modalities have the potential to improve mother’s self-efficacy through increasing feelings of competence and reflexive functioning.

## Plain Language Summary

This study was conducted by Dr Alessandra Prioreschi and her research team in May 2022 in Soweto, South Africa. The authors (Dr Prioreschi, Prof James, Prof Pearson, Ms Smith, Prof Norris and Dr Rennie) sought to 1) develop an intervention that provides personalised feedback to mothers on their infants’ movement behaviours, as well as their own interactions with their infants in an attempt to promote their confidence and bonding, and 2) test how acceptable and feasible such an intervention would be. Dr Prioreschi recruited a small sample of mothers from Soweto, and asked them and their infants (aged 4 months) to wear accelerometers (small watch-like devices which measure movement) and head-mounted video cameras in their home for a few days while playing together as they normally would. Dr Prioreschi’s research team collected the device data and converted it into easy to interpret graphs (representing daily movement behaviours), and short video clips (key moments reflecting how well mothers were doing). After presenting the mothers with their personalised feedback, Dr Prioreschi’s research team conducted focus group discussions to understand how mothers felt about the feedback and whether they found it acceptable. The mothers enjoyed receiving the feedback, and felt it would make them feel more confident. However, some issues were raised which highlighted changes that should be made to the intervention going forward. While these findings are limited to only a small sample of mothers living a poor resourced setting with socioeconomic vulnerabilities, it was able to help the authors improve the design of the intervention so that it could be repeated at a bigger scale within the Soweto community in order to test if it is actually effective. Such an intervention could benefit mothers and infants by improving caregiving practices and thus infant developmental outcomes.

## Introduction

Infant development is largely dependent on the responsivity and sensitivity of the primary caregiver. In South Africa, only 38% of children have a father residing in their home (and in poorer regions, such as Soweto where this study was conducted, only 17% live with a father), while over 90% of children under two live with their mother. Thus in such contexts the primary caregiver is usually the mother ^1^. Young women in South Africa live in social contexts which can exacerbate vulnerability, with the majority being unemployed, living with other vulnerable dependants in densely populated households, experiencing food insecurity, and reporting risk factors for poor mental health. Additionally, mothers are innately vulnerable, as having children makes women more likely to be classified as socially vulnerable ^2^. In order for caregivers to optimally support infant development, they need to feel confident in their ability to respond to their infant’s needs, and to provide a stable and supportive environment ^3^. Infants who have confident and healthy caregivers are more likely to develop resilience and emotional and cognitive competencies, to have healthy growth trajectories, and to develop healthy behavioural patterns ^4^. Socially vulnerable caregivers may find it more challenging to become attuned to infant cues ^5, 6^, engage in responsive caregiving ^7, 8^, or to feel confident that they can promote a stable environment due to the financial and emotional pressures of their daily lives and environments.

Promoting self-efficacy is thus key in the early postpartum period, especially in socially vulnerable populations, and can be achieved through sensitising caregivers to infants’ competencies and behavioural patterns, promoting healthy growth and development, and promoting positive and nurturing interactions ^9– 11^. Two strategies will be the focus of this study: 1) sensitising caregivers to infant competencies and behavioural patterns, which includes making them aware of their infant’s daily movement behaviour patterns (such as play and sleep patterns), which are in turn essential for healthy infant growth and development ^12, 13^; and 2) promoting positive and nurturing interactions using video observations of mother-infant interactions and providing guidance and feedback. Several randomised control trials have shown positive effects of strengths based video feedback of sensitive clips on parenting behaviours ^14^, yet to our knowledge none have been conducted in Africa and none have used first person perspective cameras alongside accelerometers.

Incorporating regular play time into daily routines offers an ideal opportunity for caregivers to engage and bond with their infants through stimulating activities ^15^ which encourage movement and limit restraint and in turn promote cognitive, emotional and social development ^12^. Similarly, promotion of regular sleep routines (sleep hygiene) is crucial in the development of sleep patterns in infancy ^12^, and could help caregivers to feel confident in their ability to manage their infants’ sleep. While guidelines for infant movement behaviours exist ^12^, these concepts may be difficult to implement in practice for mothers who are poorly nourished, resource strained, parenting in isolation, have poor mental health, and are likely sleep deprived themselves. As such, interventions to sensitise caregivers to infant behavioural patterns may beneficially impact caregiver self-efficacy by helping to identify opportunities for implementing changes in daily habits/routines, and highlighting opportunities for bonding. Additionally, helping mothers to understand their involvement in modelling and promoting opportunities for healthy movement behaviours for their infants ^16^ may increase their feelings of capability.

Providing in vivo feedback on infant and caregiver interactions and behaviours is an important and effective component of caregiving interventions and behaviour change interventions in general ^17^, which can help to enhance caregiving skills and thus infant outcomes ^10, 18^. In vivo feedback can include responding to behaviours with praise or criticism, or directing engagement with targeted behaviours ^18^, and can be responsive to behaviours in real-time, or following review of interactions and behaviours ^18, 19^. Promoting positive and nurturing interactions and sensitising caregivers to infant behaviours can thus be achieved through observation of caregiver-infant interactions and provision of positive reinforcement during key interactions ^20^. Providing feedback for caregivers on their responses to their infant can create space for reflexivity which can lead to changes in the caregivers’ response repertoire ^4^.

In this paper, we report findings from a pilot phase of The PLAY (Play, Love, and You) Study. The PLAY Study is an intervention being delivered as a randomised controlled trial, designed to improve infant development by encouraging maternal self-efficacy ^21^. It is crucial to pilot and test the feasibility and acceptability of intervention strategies and adapt the protocol accordingly prior to implementation and evaluation of effectiveness ^22^. Therefore, the aim of this pilot study was to 1) describe the development of, and 2) pilot and test the acceptability and feasibility of providing individualised feedback on two infant behaviours.

## Methods

### Setting and participants

Women were approached at a local taxi rank outside of Chris Hani Baragwanath Academic Hospital (CHBAH) and provided pamphlets describing the purpose of the study. CHBAH, located in Soweto, is the third largest hospital in the world, and the largest in Africa, and is financed and managed by the Gauteng Provincial Department of Health. CHBAH services patients from a large proportion of Johannesburg, as well as from further reaching areas in South Africa. Women were eligible if they 1) were >=18 years of age, 2) had given birth to a single, full-term infant in the past 4 months, 3) were the primary caregiver of that infant, and 4) resided in Soweto. Data were collected at the Chris Hani Baragwanath Academic Hospital and at participant homes over two consecutive weeks in May 2022. Ten mothers were recruited, yet only eight gave written informed consent to participate in accordance with the Declaration of Helsinki. This study was approved by the University of the Witwatersrand’s Human Research Ethics Committee (clearance number: M210846) on the 25/02/2022.

### Sociodemographic information

The participants initially completed a sociodemographic questionnaire, in which they self-reported their age, marital status, level of completed education, employment status, parity, and their socioeconomic status (assessed by self-report of the type of home they lived in, toilet facilities, access to a list of 13 potential household items, and household density) ^2^. Participants also reported their infant’s sex and age.

### Development of feedback

The feedback protocol was designed to measure and provide individualised feedback on two infant behaviours: movement behaviours and interactions with their mother. This approach was used as a means to sensitise mothers to infant routines, encourage interactive play, and promote responsive caregiving and nurturing interactions. Based on the literature described above, we believed that focussing on these areas may increase mothers’ feelings of self-efficacy.

***Sensitising mothers to infant movement behaviours***

In developing this feedback, the goal was to help participants become more aware of their infant’s movement behaviours and how these aligned with the recommended movement guidelines ^23^, how their own movement behaviours corresponded with their infant’s behaviours (i.e.: the interdependence of behaviours), and what their infants’ daily movement routines look like (for example, daily patterns of movement and sleep, when and where higher and lower movement happened, and how these patterns fit in with their goals and with the guidelines). Raising awareness to infant behaviours within the context of providing guidance may help mothers become more able to interpret their role in their infant’s development, therefore building their self-efficacy. Mother and infant movement behaviours are measured using tri-axial accelerometers and converted into visual feedback (graphs). The wrist-worn accelerometers allow daily locomotive movement to be collected at a very high resolution (100Hz) with a dynamic range of +/- 8g ( www.axivity.com) which captures all the human range of movement over a 24 hour periods for up to 10 days. This technology has been successfully applied to infant populations ^24^.

**Figure 1.**
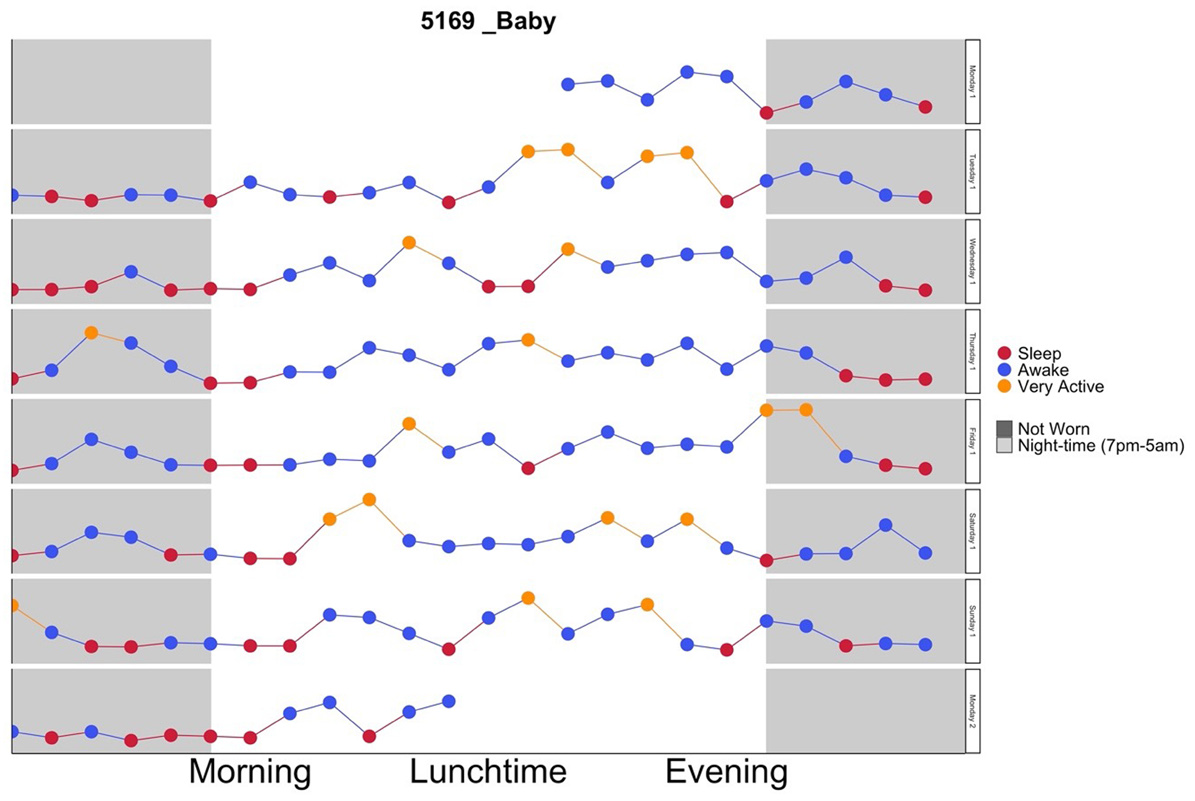
Example of infant feedback graph.

In order to formulate the content of the feedback graphs, a workshop to explore feedback theory and types of feedback on physical activity of young children and infants was held at the MRC Epidemiology Unit, Cambridge University, UK in March 2022. This workshop included authors AP and KR, as well as four other researchers with expertise in movement behaviours during the postpartum and infancy periods. During this workshop, preliminary example graphs were developed to provide a visual for participants of the infant’s movement over time. The focus was on ensuring the graphs were scientifically correct, clear in terms of the aims of the feedback messages, easy for participants to understand and visually appealing. Attendees agreed that graphical focus on daily routines, variations in routines, sleep hygiene, and peaks of high activity (potentially play) would assist with delivering the key feedback messages. From this workshop, feedback graphs were developed using a reiterative process by AS, AP and KR. It was decided from this work that the feedback would have the following format ( Figure 1 shows an example graph for reference):

- Triaxial accelerometry data (acceleration data collected in three orthogonal planes of movement) were collapsed into a summation variable that represents the volume of movement (mean ENMO (Euclidean Norm Minus One) ^25^) for hourly segments so that each point represents the overall average movement for that hour. This resolution allowed the data to be easily interpretable by the participant but still relatable to length of daily routines
- Data points for each hour were colour coded according to the intensity of movement being represented (i.e.: sleep, sedentary behaviour, light vs high intensity movement)
- All days were visualised in one combined feedback graph with each day (24-hour period) plotted on the same scale so that the patterning and routine of movement e.g. time to bed (sleep hygiene) could be clearly seen from one day to the next
- Night-time periods (between 7pm–5am) were clearly indicated with light grey shading to highlight periods of sleep or movement during this period
- Hours of the day annotated as morning, lunchtime and evening segments, which are easily understandable words and terminologies to the population group to help identify routines.
- Periods when the accelerometer was not worn were clearly indicated with dark grey shading so that it would not be misinterpreted as low movement
- The same graph format for mother’s and infants’ movement was used so that concurrent behaviours could easily be compared

***Providing video feedback on mother-infant interactions***

In developing this feedback, video recordings of mother-infant interactions were considered, and the VIPP-SD framework ^26^ was used to guide the focus of the feedback and selection of key interaction moments. A workshop was held at Manchester University, UK during two sessions in March and November 2022 with authors AP and DJ, as well as three other researchers with expertise in analysing mother-infant video data, and the psychological relationship between mental health, bonding, and mother-infant video data. Here discussions were held about what type of interactions to record, how to select appropriate footage for feedback, and how to present the feedback. It was decided that asking participants to record while playing/interacting/bonding with their infant would allow good opportunities for providing feedback. Using the VIPP-SD framework, and proposed active components for change ^14^ we decided to focus on positive fragments during the 0–4 month period. As such, key interaction moments included instances of infant smiling, mother soothing infant, mutual gaze, mother directing infant’s attention, or posture/facial expression mimicking. These moments were chosen to highlight positive fragments of an interaction in order to promote mother’s feelings of self-efficacy and to reduce negative emotional responses. Proposed key interaction moments for feedback in older infants would include perspective taking, recognising child feedback, empathy for child’s needs, and reflexive functioning ^14^ – yet these were not tested in the current study. It was decided that all feedback videos should be of similar length (approx. 30 seconds), should end on the peak positive moment of the interaction, and should be played at least twice, pausing and repeating important moments or transitions to help mothers recognise child signals ^14^.

### Pilot testing of feedback

***Feasibility of delivering the feedback***

A week prior to the feedback delivery, mothers and their infants were asked to wear an accelerometer (AX3) in a watch-like wrist band for at least four full days (morning and night) while continuing with their normal daily activities. The AX3 monitor records second-by-second changes in acceleration in three planes of movement from the limb to which it is attached providing an indication of bodily movement over time (i.e.: transitions from periods spent in sedentary behaviour to light intensity movement). Mothers and their infants were also required to wear a small, head mounted camera (“headcam”) for at least three, five minute sessions over the course of four days while interacting with their baby. Participants were instructed to spend these five minutes playing, bonding or engaging with their infant as they normally would. The headcams provided a first person perspective of the interaction from both the mother’s and the infant’s perspectives.

Upon collection of the devices, data were downloaded and feedback plots and videos were created. Accelerometer data were downloaded using Omgui software, and the raw 100Hz data were processed to scan and fix any recording errors, to resample the signals to a uniform 100Hz signal by linear interpolation, and to calibrate to local gravity using a well-established method ^25, 27^. Periods of nonwear were identified as windows of >=60 mins where the standard deviation of acceleration in each of the three axes was <13mg. The acceleration intensity metric Euclidean Norm Minus One (ENMO) was derived from the vector magnitude from the three axes ((X, Y, Z: X2 + Y2 + Z2) ^0.5^). These validated processing python scripts were performed in an executable application WAVE (waveform accelerometry processing software) created by the MRC Epidemiology Unit at Cambridge University, UK ^28^. The processed data was then analysed in STATA SE V17 for Mac (free alternative – RStudio) to collapse into hourly mean ENMO values, and RStudio V14 used to convert the data into feedback plots, where individual participant (mother and infant) data were graphically presented over time. Time spent in different levels of acceleration were illustrated that broadly represent time not moving (e.g. sleep), awake in light intensity activities and being very active for infants based on previous data in infant populations ^24^; and time spent sedentary (<50m *g*), light (50–110m *g*), and in moderate to vigorous (>110m *g*) activity for mothers. The cut offs for the mothers activity intensity levels have been developed in adult populations ^29^. Processing of the accelerometer data and creation of the graphs was designed to be relatively automated requiring minimal input or expertise, and data could be batch processed. Therefore downloading and processing the data took about 15 minutes per participant, while producing the feedback graphs took less than 5 minutes per batch. An example graph is presented in Figure 1.

Headcam video files were downloaded and initially reviewed to identify eligible videos (where lighting was acceptable, and both mother and infant were in view in the respective videos). Thereafter, key interaction moments were selected. Once a key moment was identified, the two videos (mother and infant perspectives) were placed side-by-side and synced using Adobe Premier Pro 2022 (free alternative – DaVinci Resolve). The side-by-side clip was trimmed to show only the lead up to the positive moment, and videos lasted approximately 30 seconds each. Producing the video feedback included downloading the data (5 minutes), sorting through the data to find the matching mother and baby perspectives (approximately 5–10 minutes per video recorded), synchronising the videos (5 minutes per video pair), and choosing the positive moment to feedback (10–15 minutes per synchronised video). Thus the total time taken was dependant on how many videos the mothers recorded and the duration of those videos.

During the feedback session, which occurred within three days following collection of devices, mothers were taken to a private room at our research offices in the Chris Hani Baragwanath Academic Hospital, and feedback was delivered by a research assistant who was trained in the feedback methodology, but had no specific psychological or behaviour change training. The sessions were supervised by AP. Mothers were first presented with their infant’s graphs and then their own graphs. Graphs were explained by highlighting the time segments on the x axis, the rows for each day of recording, and the colour coding indicating the intensity of movement at each hour of the day. Feedback was given around how active they were in general, daily routines and peaks in movement in relation to those daily activities (i.e.: mother was asked to recount and contextualise peaks in movement within the day to understand what behaviours caused those peaks), sleep routines and duration as well as effect on movement the following day, correlations between mothers’ and infants’ movement behaviours, and infant nap times. Mothers were then presented with the 30 second video clips of their interactional moments. Some mothers were shown a still image from the video which encapsulated the key moment for feedback, followed by the full video; whereas for others this order was reversed. Videos were replayed at least twice, and paused at key moments. Feedback was provided by focussing on positive fragments with the aim of increasing mothers’ feelings of confidence ^19^.

***Acceptability and perceptions of receiving feedback***

Prior to receiving feedback, a group discussion was held to discuss mothers’ perceptions of and goals around their infants’ movement behaviours as well as their own movement behaviours. Guidelines for mothers ^30^ and infants ^12, 23^ 24-hour movement behaviours were presented, whereafter the discussion was focused on 1) mothers’ awareness and understanding of these guidelines, 2) mothers’ goals and the support they felt they would need, 3) daily routines and how important mothers felt these were, and 4) what mothers would like to know about their own movement behaviours and their infants’ movement behaviours. Mothers were also asked to discuss a day in their life with their infant. They were encouraged to share their favourite moments with their infant during the day. Midway through the discussion, mothers were asked to complete a self-efficacy questionnaire ^31^, whereafter they were asked to discuss areas where they felt they needed more support, and to share some goals related to their self-efficacy. This session was audio recorded and transcribed and translated where necessary.

Directly after receiving the feedback (all mothers received feedback on the same day), a final group discussion session was held, whereby mothers discussed their movement behaviour and interaction video feedback as a group, linked this feedback to their initial goals discussed, and provided any other general thoughts about the feedback acceptability. They specifically focused on what they liked or did not like, how helpful and important they found the feedback, and whether they could link their feedback to their interactions with their infant in general (relatability). At this time, mothers were also asked to repeat the self-efficacy questionnaire and to identify and discuss any perceived changes compared to the first instance. This session was audio recorded and transcribed and translated where necessary.

Mothers were then asked to complete a questionnaire assessing the acceptability of the feedback in terms of usefulness, clarity, relatability, as well as feedback delivery options. This data was blinded by using numerical identification numbers in an attempt to reduce social desirability bias. Based on the theoretical framework of acceptability proposed by Sekhon *et al*., ^32^ we aimed to use the qualitative and quantitative data to determine perceived affective attitude, burden, ethicality, intervention coherence, opportunity costs, perceived effectiveness, and self-efficacy.

### Analyses

Sociodemographic information, acceptability data and self-efficacy data were summarised and presented as means (SD) or N (%). Absolute and percentage change in self-efficacy total scores and individual item scores from before to after the feedback session were then calculated and presented.

Two trained members of the research team facilitated the focus group discussions (FGDs). One member was fluent in the vernacular and the other took notes on the observed process which would supplement the information gained by the audio that was recorded in the immersion-crystallisation approach ^33^. Notes and processes were assessed after the first FGD, and this informed the discussion guide for the second FGD. FGD sessions were audio recorded, and later transcribed and translated verbatim. Each FGD was analysed using an inductive, iterative analytical approach ^33^. Main emerging themes were identified by coding transcript for content, line by line. Quotes were then categorised into themes and subthemes. The themes and subthemes from both FGDs were then combined, and restructured and condensed until no new themes emerged (analytical saturation) and all authors were in consensus that acceptability could be determined. These results were consolidated, and patterns in the data were interpreted and described according to themes and subthemes, and with presentation of exemplar quotes.

## Results

### Feasibility of the feedback

Mothers included in this study were generally single, unemployed, and less than half had completed formal schooling. Most mothers had two or more children, and lived in informal housing (such as shacks and small, temporary prefabricated structures) with only outdoor toilet facilities available. Data is presented in Table 1.

**Table 1.** Sociodemographic information.

		Mean (SD)	N (%)
Age (years)		30 (9)	
Marital Status	Single		4 (57)
	Married		1 (14)
	In a relationship		2 (29)
Employment status	Employed		0 (0)
Education	Secondary school		4 (57)
	Completion of primary education		3 (43)
Infant age (months)		2 (2) range 1-4	
Infant sex	Female		3 (43)
Parity	1		1 (14)
	2/3		4 (57)
	4/more		2 (29)
Household density (people/room)		3 (3) range 1-8	
Type of home	Shack		2 (29)
	Zozo *		5 (71)
Toilet facilities for home	Indoor		2 (29)
	Outdoor		5 (71)
Socioeconomic status score (/13)			8 (2)

*Zozo in South Africa refers loosely to any prefabricated or temporary building, especially a small one

***Compliance***

All of the mothers and infants wore the accelerometers for a sufficient amount of time to provide enough data to analyse and be able to provide feedback (>= 4 days). Feedback graphs were therefore available for 100% of participants. Processing of the accelerometer data and creation of the graphs was designed to be relatively automated requiring minimal input or expertise, and data could be batched processed so that it would be scalable. Similarly, all of the mothers and infants wore the headcams at least once providing matching videos of 3 minutes or more from both perspectives that could be synchronised, and so feedback videos were available for 100% of participants. Feedback on mother-infant interactions focused on positive fragments including one or more of the following: infant smiling (four instances), mother soothing infant (one instance), mutual gaze (four instances), joint attention on an object (one instance), or mimicking facial expressions (three instances).

### Acceptability of the feedback

Analysis of the FGDs revealed themes related to maternal perceptions about routines, play and movement guidelines, self-efficacy, and bonding; as well as themes related to the acceptability of feedback sessions. These themes are discussed below in conjunction with the results from the acceptability questionnaires, and exemplar quotes are presented in text and in Table 2.

**Table 2.** Exemplar quotes from the focus group discussions.

Maternal perceptions of infant movement patterns, and their own self-efficacy	
**Routines**	“ *For me it is important because I handle a lot on my own, so if I am sleeping for me I am resting my mind*”
	“ *the baby and I sleep the whole day … I bath the baby but if I feel like I am lazy and I bathed the baby yesterday, I * *won’t bath them again today*”
	“ *I hardly think of routines because my baby and I are always sleeping, we don’t have a set routine so we just sleep* * the whole time*”
**Play and movement** ** guidelines**	“ *But some of us when we are really in a hurry we feel like we are going to faint*” [moderator *: “You shouldn’t be* * doing it for long, just 30 minutes”*] “ *Then I will faint*”
	“ *..so if my baby and I didn’t sleep well in the morning…. we will then sit in bed in the mornings and hence my* * activity will show red* [inactive] *because that means that* [we] *are lying in bed watching something on the phone.* * But being busy means I have to wait for her to sleep … because she doesn’t like if I am not holding her… then once* * she gets tired, she dozes off to sleep and that is when I can do my things because she’s sleeping, but it gets tiring…*”
	“ *Yes, I try to have time to play with the baby but there really isn’t any*”
	“ *Maybe the sound of the music from the cartoons also helps somehow*”
	“ *I also* [thought] *that cartoons were a good thing too because the baby would jump up and down and be excited* * while watching the TV… Yes, the baby plays and jumps around*”
**Bonding**	“ *But my favourite time is playing with him*”
	“ *For me it is early mornings before the other kids go to school and then I sing to the baby and we dance*
	“ *When I bath her and change her, she will be active and happy in the bath, when we are in the water she doesn’t* * want to get out and she makes noise and for me that is a connection, so after bath time too*”
	“ *But we connect mostly after bath time*”
	“ *So nappy time is the best time for me because we bond and then also in the afternoon, that is my best time”*
Acceptability	
**Movement behaviour ** **– mothers feedback**	“ *it showed how much time we need to put aside for playtime and all the other thing*s”
	“ *I didn’t know you should play with the baby because I would up and leave and not play with the baby so that was * *useful for me*”
	“ *I learnt a lot of things like the baby having a sleeping routine*”
	“ *some of us didn’t know the things that we read about so when it comes to routine, it will help with setting it up* * and learning more*”
	“ *I also thought it was a good thing to learn to be active so you are not always thinking and worrying*”
	“ *There was a lot of information*”
	“ *I sleep a lot which is not good for my vitals and my mental health, one needs to be active and exercise*”
**Mother-infant** ** interaction– mother’s** ** feedback**	“ *What I liked from the videos is when the baby showed her tongue*”
	“ *We tend to believe that when you are playing with a baby they don’t concentrate but in fact they are concentrating* * that is what the video showed us, that the baby is concentrating like we saw on the video. And sometimes I get sad* * thinking that which is not true*”
	“ *I thought I wasn’t good at playing with the baby, but I realised that when I laugh the baby laughs and it was one* * of the things that I had ticked that I then thought I couldn’t do. So now the baby laughs and I am also able to calm* * them down*”
	“ *And that when I am talking, the baby focuses on me*”
	“ *I always wondered if my baby is able to feel my love for them*”
	“ *I learnt I have to be more patient with the baby*”
	“ *For me the issue is the phone, I can’t play-play with the baby so what I do is, I go to YouTube, play some cartoons* * and music so to me that is how I play with my baby and not realising the phone is a bad thing*”
	“ *What I didn’t like was that the baby didn’t look entertained while in fact it was*”

***Maternal perceptions of infant movement patterns, and their own self-efficacy***

**Routines**

There were mixed feelings about whether routines were important, or featured in mother’s lives with their infants. Some mothers said that routines were important to allow them to have time to rest or get on with other household duties:“… *once she sleeps I can clean then bath then she is up the day goes on then…*”. Two mothers mentioned that routines were irrelevant to them at this early postpartum stage as they were always sleeping . One mother suggested that if she could be taught about routines, and how to practice routines she may benefit, but that currently some mothers were not interested in knowing about routines: “ *some of us don’t even read or want to know what a routine is all about, we just see it as something as useless*”. For those mothers who practiced routines, feeding times and sleeping times seemed to be most important.

**Play and movement guidelines**

Most mothers were not aware of the movement guidelines for adults, or infants. When presented with the recommendation that adults should do 30 minutes of moderate to vigorous activity per day, some mothers did not believe this was possible, yet others believed they could achieve these guidelines through daily activities such as washing dishes and walking down the stairs. One mother said that she could not meet the guidelines because of the demands her infant placed on her: “ *because she doesn’t like if I am not holding her… then once she gets tired, she dozes off to sleep and that is when I can do my things because she’s sleeping, but it gets tiring…*”. Regular reference to activity causing fatigue may indicate that in this context, where mothers are already living with limited resources and food insecurity, inactivity may be a way to conserve energy. When considering the physical activity/play recommendation for infants, most mothers said that they did not have time to play with their infant, and one suggested that counselling may be helpful in this regard: “ *I also need counselling…I don’t have a lot of time for the baby because I am always stressed … sadly I don’t have time because I am very busy”.*

When considering the physical activity/play recommendation for infants, most mothers said that they did not have time to play with their infant “ *Yes, I try to have time to play with the baby but there really isn’t any*”, and one suggested that counselling may be helpful in this regard “ *I also need counselling…I don’t have a lot of time for the baby because I am always stressed … sadly I don’t have time because I am very busy”.*

When considering the sleep guidelines for infants, one mother commented that her baby was always sleeping, while another said her infant only “ *…sleeps two minutes*”. When considering the TV time guidelines for infants, many mothers were surprised to learn that TV time is not recommended in the first two years of life. Most mothers commented that they thought that TV, and cartoons specifically, were beneficial for infant’s development: “ *I thought TV was good too… I watch movies on my phone too and the baby ends up watching and laughing. I thought that it was a good thing because the baby needs to learn to look at things and see things clearly and learn to hear sounds so I thought that was helping with the child’s development”*. These quotes indicate that mothers allowed screen time as a means to improve development, which, while in contrast to scientific evidence, does indicate that mothers’ motivation in their behaviour was linked to their desire to help their infants learn and progress.

**Self-efficacy**

Issues discussed by mothers while completing the self-efficacy questionnaire included not knowing how to make the infant happy, not knowing how to soothe or calm the infant when they are crying or upset, that the infant is constantly crying, not understanding what the infant wants, not knowing if the infant is responding well to the mother, and not knowing if they were doing the right thing. One mother summarised these concerns saying: “ *a lot of people don’t know how to calm the baby down, they don’t know what the baby wants when she is crying and you are not sure maybe if you are making baby happy*”. One mother reported finding her child irritating, and another that she struggled to show affection to her baby.

**Bonding**

When asked about moments where mothers felt bonded to their infants, or favourite moments during the day, many mothers spoke about breastfeeding as a special time: “ *Breastfeeding for me when I am breastfeeding I forget about all my stress then I am able to focus on the baby and it helps me calm down a bit*”. Mothers also spoke about play time as a time when they felt connected to their infant; two mothers specifically enjoying time playing together when all their children were home from school: “ *From 1 to 1.30 we are playing, the other kids are back from school and we all play together and we connect doing that*” *.* Three mothers mentioned enjoying bath time or changing their infant as a moment for connection.

***Acceptability***

**Movement behaviour – mothers feedback**

After receiving the feedback, mothers were asked to comment on how they found receiving the information. All mothers said they found the feedback session useful. Specific reasons for finding it useful were largely related to learning things they did not know before, such as the need to make specific time to play, the importance of sleep routines and routines in general, and the importance of keeping healthy and active: “ *I didn’t know all these things… they were all new to me*”. When asked what else mothers may need to support them regarding movement behaviours, a few mothers mentioned needing more time, counselling, and help in general. When mothers were asked if there was further information they would like to receive, most asked for more information about how to get their infant to sleep more/better.

Acceptability questionnaire data on the movement behaviour feedback is presented in Table 3. Most mothers reported that they completely understood the feedback, and found it very clear and very informative. All mothers found they could relate the graphs to their infants’ day-to-day behaviours, found the graphs useful to their parenting goals, and felt the graphs would help them understand and improve their infant’s behaviours a lot. Some mothers elaborated while completing the questionnaires, and this data is presented in Table 4.

**Table 3.** Movement behaviour feedback acceptability.

Impressions of feedback		N * (%)
Understanding		
	Mostly/Little/Do not understand	0 (0)
	Understand a lot	2 (29)
	Completely understand	5 (71)
Clarity	Not clear enough	0 (0)
	Clear enough	1 (14)
	Very clear	6 (86)
Informative	Not informative	0 (0)
	A little bit informative	1 (14)
	Very informative	6 (86)
Delivery	Graphs alone	3 (43)
	Graphs and a phone call	3 (43)
	No preference	1 (14)
**Baby Feedback**		
Relating to day-to-day activities	Not at all/A little	0 (0)
	Very well	7 (100)
Which behaviours were easy to relate	Sleep and wake times	6 (86)
	Naps	7 (100)
	Feeding	5 (71)
	Play times	4 (57)
Were the graphs useful according to your parenting goals?	Yes	7 (100)
How much will these graphs help you with understanding and improving baby’s play activities	A lot	7 (100)
Delivery	App	5 (71)
	WhatsApp	3 (43)
	Phone call	1 (14)
	Face-to-face	2 (29)

*Only 7 of the 8 participants completed the questionnaire as one had to leave early

**Table 4.** Comments on feedback acceptability and percentage change in self-efficacy by individual participant.

Mother *	Comments on movement behaviour feedback	Comments on mother-infant interaction feedback	Percentage change in self-efficacy
1	I liked the way the video was clear and that I was doing very well yet I didn't think I was.	My baby is active so I'm very happy. The graphs show that my baby has good routine in terms of sleep and wake up times. I am not happy about my activity. The graph doesn't have even a single orange dot (moderate activity). After explaining to me about the graphs I was able to see when I am asleep or when I am doing something	7,14
2	I felt discomfort when I wasn't sitting very well and ashamed at seeing how things are in my house. I felt like everyone was looking at the house instead of the videos	The red dots means the baby is asleep and orange means very active. The graphs showed that I was not very active most of the time but on Thursday I was a bit active as I was during laundry	18,64
3	I liked the video but not much since the baby was not her usual self. she was not happy having a camera attached to her	After the feedback sessions I understood the graphs well. My baby for examples sleeps a lot so I was advised to change that I was shown that I slept too much and was very active at night when I should be asleep	0,00
4	I liked everything about the video	I saw that my baby doesn't sleep at the same time so I need to make some adjustments to this regard. They said do a lot of movements but I’m not active [enough]. I am very busy at home with cleaning and in the kitchen	21,52
5	I saw where I need to improve and what to do if I need to get attention from the baby.	Red dots baby is sleeping. I can see when I'm feeding my baby	15,71
6	I was happy to see how my baby responded to my interaction efforts i.e. trying to make her laugh	I can relate by the colour codes. [Specifically], sleeping since the graphs show that when the baby is sleeping I am usually awake	
7	The baby was relating [to me] when I was busy with my phone checking time so I learnt that I should try not get distracted when I am with my baby	Orange dot shows that my baby was very active Tuesday and Wednesday. Red dots showed that my baby was asleep most of the time on Thursday since he wasn’t okay. Most of the time I would walk to the shops, so I saw that in the graphs	2,74

*Only 7 of the 8 participants completed the questionnaire as one had to leave early. Only 6 of the 8 participants completed the self-efficacy questionnaire after receiving the feedback

**Mother-infant interaction**

After seeing the interaction moments mothers commented on memorable parts in the videos such as those when the infant was being cute. Mothers also spoke about moments in the videos where they could see how their infant was responding to, or engaging with them as this indicated moments of bonding: “ *I liked the part where, when I play with the baby, her eyes follow the object I am holding so that means I am able to entertain her and we are somewhat bonding*”, or moments that showed mothers that they were doing better than they initially thought: “ *I thought that I couldn’t play with my baby but after watching the videos, I realised I can play with my baby and make her laugh*”. Many of these responses indicated an improvement in the mothers’ confidence and self-efficacy. Some mothers also discussed feeling that they had learnt something from watching the videos, such as patience, or avoiding excessive use of devices. Some mothers reported on things they did not like seeing on the videos, indicating that the videos showed unfavourable moments or did not represent how they believed their infant truly felt in the moment: “ *What I didn’t like about the video was that the baby didn’t laugh as usual and looked irritable so that is what I didn’t like*”. One mother indicated that she felt the person delivering the feedback did not focus on moments showing aspects of the infant’s behaviour that the mother had identified as important: “ *And another thing that she* [person delivering feedback] *didn’t do is pay attention to how entertained the baby was*”. One mother mentioned that she had nice videos of her and her infant connecting that were not captured on the headcams. This indicated that mothers wanted their strengths to be noticed, and did not appreciate any misinterpretation of their interactions or indications of weaknesses as they perceived it.

Regarding the cameras themselves, and the process of recording their interactions, one mother indicated that at first her infant was “ *irritated*” by the camera, but had adjusted by the second attempt. Some mothers reported contextual factors that impacted their ability to record videos, for example one mother mentioned that she had to record videos while she had “ *personal problems*” going on, and another battled with not having electricity while trying to record videos, which affected lighting. However, regardless of these contextual barriers they were still able to record some videos and receive feedback. Only one mother reported that it felt “ *unnatural*” to record the videos because she felt “ *forced*” to play with her baby, when normally she would “ *sit and look at* [her] *phone the whole time*”, yet this indicated that she found the time to play with her baby when normally she may not have done so.

Acceptability questionnaire data for the mother-infant interaction feedback is presented in Table 5. All mothers reportedly enjoyed receiving the feedback, felt that the feedback would help them as a mother, felt that the feedback related to their goals as a mother, and felt that the feedback would help them understand and bond with their infant better. One mother mentioned that she would like to better understand what her infant’s cues and behaviour means, while another expressed that she hoped to see herself improve in the future. Some further acceptability points mentioned while filling out the questionnaire are included in Table 4.

**Table 5.** Mother-infant interaction feedback acceptability.

Impressions of feedback		N * (%)
Did you enjoy receiving feedback?	Yes	7 (100)
Which video format did you prefer?	Mom and baby side-by-side	6 (86)
	Baby only	1 (14)
	Mom only	0 (0)
	All three	0 (0)
Which order would you like to view the feedback?	Video first, then photo	6 (86)
	Only video	1 (14)
	Photo first, then video/ Only photo	0 (0)
How informative were the videos?	A little bit informative	1 (14)
	Very informative	6 (86)
Delivery	Videos alone	2 (29)
	Videos and a phone call	4 (57)
	No preference	1 (14)
How much will receiving this feedback help you as a mom?	A lot	7 (100)
How much does this feedback relate to your own goals as a mom?	A lot	7 (100)
How much do you think this information will help you with understanding your baby and improving the bond between you and your baby?	A lot	7 (100)
Delivery	App	3 (43)
	WhatsApp	4 (57)
	Phone call	0 (0)
	Face to face	0 (0)

*Only 7 of the 8 participants completed the questionnaire as one had to leave early

In terms of delivery of the feedback, mothers enjoyed seeing themselves and their baby side-by-side and most mothers wanted to see the video first, followed by a still image of the key moment. We asked about mechanism of delivery in order to determine whether providing digital feedback would be feasible in this context. Most mothers wanted the feedback delivered via WhatsApp or the PLAY Study app along with a phone call, and most indicated that they would need the app to be data free.

***Perceived effectiveness based on the self-efficacy questionnaire***

**Figure 2.**
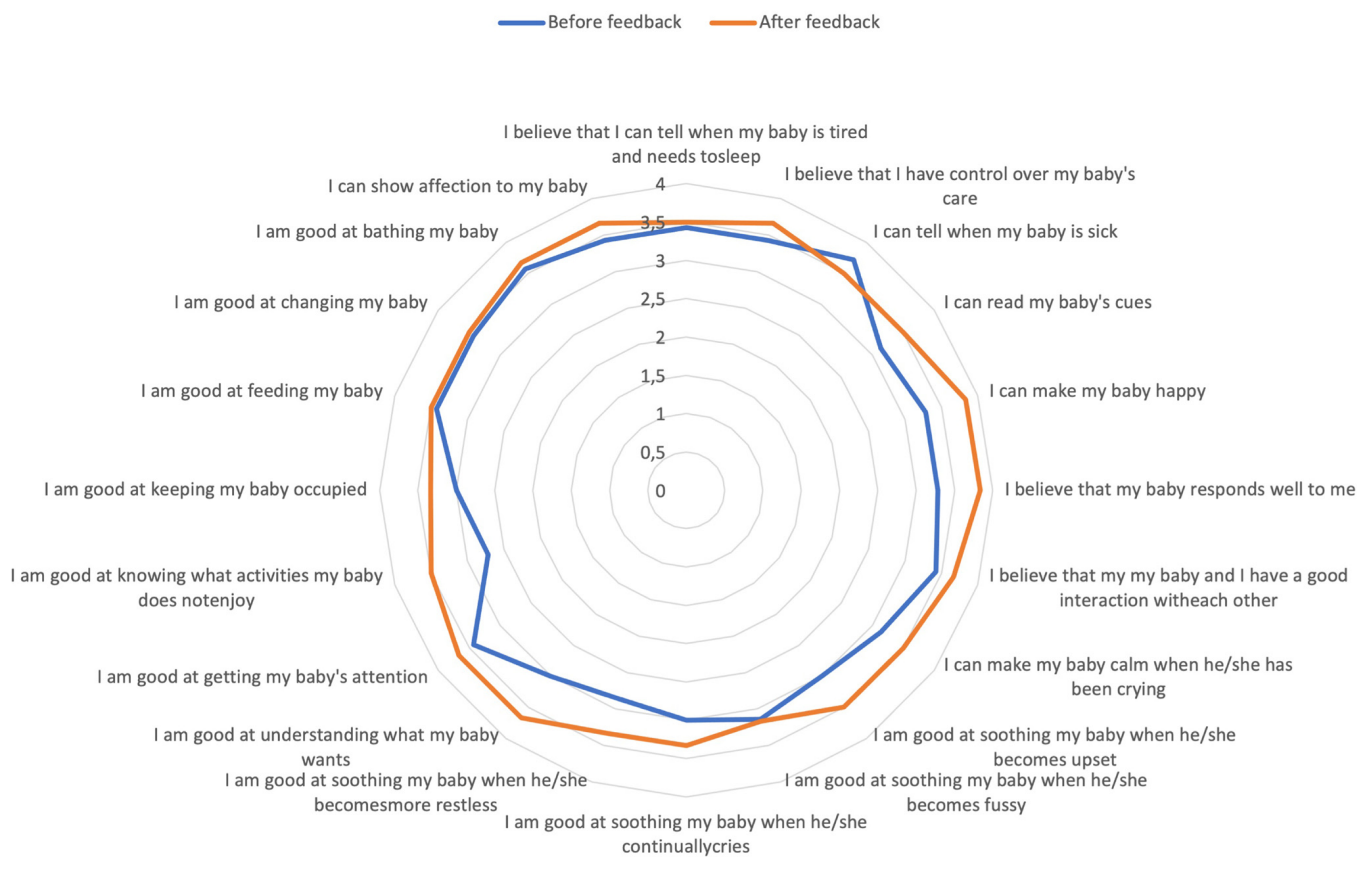
Self-efficacy individual item scores before and after receiving feedback assessed using the Perceived Maternal Self-Efficacy Tool (PMP S-E).

Prior to, and after mothers received the feedback, they were asked to complete a self-efficacy questionnaire and to discuss their feelings of self-efficacy based on the question items. While this data cannot be interpreted as actual effectiveness, while completing the self-efficacy questionnaire for the second time some mothers spoke about feeling “ *Confident*”, and “ *Confident in knowing that I am trying*”. Out of interest we have reported change in self-efficacy individual item scores before and after receiving the feedback in Figure 2, and percentage change in total self-efficacy score for each individual participant is shown in Table 5. In total, self-efficacy scores improved by 8.4% after receiving the feedback. Questionnaire items which improved the most included knowing what activities baby does not enjoy (22% increase), being good at understanding what baby wants (18% increase), being able to make baby happy, believing baby responds well to mother, being good at soothing baby when they become restless, and being good at soothing baby when they become upset (all 14%). All items improved after receiving feedback except for one - being able to tell when baby is sick - which decreased by 6% following feedback.

## Discussion

The study aimed to describe the development of, and test the feasibility and acceptability of two individualised behavioural feedback modalities provided to mothers of infants in a socially vulnerable context, with the hope that providing such feedback in a supportive manner could improve maternal self-efficacy. The hypothesis behind the development of this feedback was that providing behavioural feedback to socially vulnerable mothers, when embedded in supportive intervention approaches, can help change mother’s perceptions of themselves and their infants thus increasing self-efficacy; and that more positive, self-actualising narratives of mothers will ultimately promote infant development.

The process of developing the feedback involved expert consultation and discussion groups, followed by iterative feedback development and design until all authors agreed that the feedback methodology was evidence based, and supported key messages for behaviour change specifically in the context of parenting. Upon implementation of the feedback in this pilot sample, we found that participant compliance with using the measurement tools was 100%, and that we were able to develop feedback on both behaviours in all instances. Furthermore, we were able to deliver the key messages hypothesised to promote change in a supportive manner. This showed that implementing the feedback methodology was feasible in this context. The feasibility of delivering video feedback to parents with infants has also been shown in other contexts, with various intervention aims, and with different population groups ^34– 36^. However this is the first study we are aware of to deliver visual feedback on infant movement behaviours.

In testing the acceptability of the feedback, we were able to determine whether the development was successful in making the feedback easy to understand, relatable for mothers in terms of their lived experience with their infants, and whether the feedback was likely to be effective based on our hypotheses. Based on the seven constructs of acceptability ^32^, we found that affective attitude, intervention coherence and self-efficacy were good, with participants finding the feedback clear, useful, and easy to understand and engage with, which is essential in this group of mothers who have a low socioeconomic status and are largely uneducated. Furthermore, delivery of feedback via a lay person trained in the feedback methodology did not seem to affect acceptability. This population of socially vulnerable mothers is often neglected when it comes to public health services, resources and support ^2, 37^. Therefore creating a tool which is relatable and simple to relay in this population, without the need for advanced skills or lengthy training, increases the likelihood that such feedback could be implemented on a wider scale. However, mothers did indicate that they would appreciate more information in order to learn more. A resource provision component of the intervention would likely improve affective attitude, and more education could be added to the feedback sessions. The PLAY Study trial does include an education component, by provided content and resources that align with the feedback via an app ^21^.

There was some perceived burden to participation in the feedback intervention specifically in relation to the use of the cameras, which could be difficult to use on infants, and was affected by contextual factors such as lack of electricity affecting indoor lighting. Mothers also reported finding it difficult to prioritise recording a video. This indicates that the burden of using the measurement devices needs to be considered, and mothers should be provided with plenty of time to record a video without feeling pressured. Thorough training on the use of the cameras, and ways to ensure proper lighting within the context of this setting should be included in the data collection procedures. The burden of using these measurement tools may impact the scalability of such an intervention, and this should be further considered in the evaluation of the PLAY Study trial following implementation. While opportunity costs were difficult to assess in this pilot phase, which was only one week long while the PLAY Study trial is 12 months long, one mother reported having to act differently to normal by playing with her baby, while another reported feeling uncomfortable that the viewer would see her home in the videos, and also did not feel comfortable in how she herself appeared. Another mother felt that the person delivering the intervention did not focus correctly on the infant’s behaviour, or misinterpreted the infant’s behaviour. While first person observation has been shown to be less intrusive and to allow for more natural free-living footage than other methods of observation ^38^, it must be considered that some interactions may not feel natural to mothers, and therefore may not deliver a relatable message. If mothers feel embarrassed or self-conscious while receiving the feedback, or if they feel like the person delivering the feedback is not correctly interpreting their interactions with their infant, they are unlikely to incur benefit from the feedback session. Thus delivery of the video feedback must be done in a sensitive manner and must focus on a strength based moment, and training on how to interpret behaviours as strengths, while still being open to mother’s own perceptions of the interaction, must be provided. Expert input may be needed in some instances. While mothers enjoyed receiving the feedback on their movement patterns, many were left feeling disappointed in their own lack of physical activity. Physical inactivity is extremely common in South African women ^39– 41^, and women are known to be even less active during the postpartum period ^42^. Mothers asked for more information on how to improve adherence to movement guidelines, or how to develop better routines with their infant. Therefore, in order to ensure that the movement behaviour feedback is successful in improving maternal self-efficacy (rather than leaving mothers feeling unsure of how to improve), it needs to be provided in the context of a supportive intervention, which should include provision of health literacy resources and personalised guidance.

Ethicality was good in the sense that participants found the feedback relatable and in line with their parenting goals. They specifically found the aspects related to bonding and promoting self-efficacy important and were interested to learn more about the importance of movement behaviours and routines. Even though mothers indirectly identified routines as important for bonding, when asked specifically about routines there was a sense that routines are not the norm in this context and potentially not seen as a relatable or relevant construct. This indicates a potential mismatch in our framing of routines in comparison to mothers’ conceptualisation of parenting. Regardless, mothers remained invested in the movement behaviour feedback sessions, and still perceived benefit from them; indicating that the process of providing feedback along with guidance in a supportive context was helpful in normalising the concept of, and adoption of routines even when not aligning directly with mothers’ initial preconceptions.

Mothers perceived that the feedback was likely to be effective in supporting their parenting goals, improve bonding with their infant, help them understand their infant’s routines and behaviours, and help them in being a mother. From the perceptions of the mothers following implementation of the feedback delivery, we were able to determine various factors that may point to the potential for the feedback to be successful in improving self-efficacy, and this reinforced the decisions made in the development of the feedback. For example, when discussing moments where mothers felt bonded to their babies (and therefore were more likely to be feeling confident in their abilities), mothers referred to periods of time set in daily routines, such as bath time, feeding time, nappy time, and play time. This reinforced our hypothesis that focussing on sensitising caregivers to infant behavioural patterns and making them aware of their infant’s daily routines through the use of the accelerometry derived movement graphs, may highlight opportunities for bonding and for building mothers’ feelings of competence; and that dividing the data into time based segments could help mothers identify these routines. Mothers were also able to see the relationship between their own movement and the movement of their infant at an hourly level. Showing mothers that their physical actions had a direct impact on their infant may provide mothers with a sense of their ability to affect change, even in an environment where they may feel they have no power due to lack of resources and information.

We also found that the strengths based approach to delivering feedback seemed to help mothers feel more bonded, and realise that they were doing well based on viewing the video footage of these positive moments. Mothers were able to derive confidence from viewing footage of positive moments that may have gone unnoticed during the real-time occurrence of the interaction. For example “ *We tend to believe that when you are playing with a baby they don’t concentrate but in fact they are concentrating that is what the video showed us... And sometimes I get sad thinking that which is not true*”. This strengths based evidence gave the mothers something they could reflect back on to realise they did in fact have what was needed to provide a developmentally rich environment for their infants, thus hopefully planting the seed that they could do a good job of parenting regardless of their economic and social environment. For example, there was a shift evident from mothers relying on providing entertainment and teaching their infants through the use of screens, to realising that they were able to entertain, engage and help develop their infant themselves without external devices/resources. This indication that mothers were learning something new shows that what was being learnt was of importance to the mother and her infant, and may therefore be likely to cause change in her behaviour.

Most mothers reported learning from the feedback and feeling more confident following the feedback session, indicating that likely mechanisms for change may be through increase in skills, feelings of competence, as well as reflexive functioning ^14^. There was less evidence for changes in empathy, recognizing infant perspectives or recognition of infant intentions that may come with feedback at later stages of development– which is in line with the proposed key interaction moments for feedback in older infants decided during the development process, but not tested in the current study. Since maternal-self efficacy is so strongly linked to responsive caregiving, it is likely that if effective, such an intervention could ultimately improve infant development and overall well-being ^4, 9^. Studies have shown that parenting interventions are most effective when requiring less investment in terms of time (fewer sessions) and money, and when focusing specifically on parental sensitivity ^43^. This is important in an under-resourced context such as South Africa, where public health interventions and policies are not well funded or implemented ^41, 44, 45^; yet health disparities are high. Children in South Africa, like many other lower income settings, are suffering from limited opportunities for optimising development ^1, 46^, as well as poor general health and well-being, including a high prevalence of stunting, overweight and obesity ^47^, and exposure to childhood adversity and violence ^48, 49^. Additionally, the majority of these children are being raised by single mothers ^1^. Regardless of resources, mothers are innately vulnerable ^2^, and so supporting mothers in the first few months of their infant’s lives may be the key to reversing some of these disparities, even as contextual issues continue to impact on early childhood development.

We achieved 100% compliance, even though there were some aspects mothers did not like about each modality of feedback. This indicates that mothers were able to trust the delivery team in viewing their homes and observing and discussing intimate, potentially vulnerable moments with their infants; and that they perceived benefit even when they reported acceptability concerns. It is important for future translation to reflect on which characteristics of the team, and principles used during delivery created this trust. Based on previous findings from qualitative research about how best to deliver health interventions to women in Soweto (data not published), we were careful to choose a relatable female to deliver the feedback, who was a similar age to participants, could speak in the vernacular when necessary, and who had experience raising her own children – yet was not from the same community as participants and would not be seen as someone who may know others within their community. Feedback delivery was overseen and assisted by the principal investigator (AP; also a female of similar age to the mothers, yet not from the same community or economic or social context), the team always wore study and hospital branded uniform, and feedback was delivered in a private room within the hospital, which may have provided a sense of the feedback being evidence based and medically sound. The person delivering feedback was thoroughly trained in the strengths based approach, and the proposed mechanisms of change; and was closely guided using discussion notes and key topics for conversation following review of the footage prior to delivering feedback. Lastly, we focused at all times on being professional, treating the women with respect, sensitivity and kindness, and on trying to show them that they are the experts when it comes to their own infants.

This pilot study has highlighted the importance of testing the acceptability of interventions ^32^, and involving participants in the process of developing and refining interventions ^50^ prior to implementation. However, it is limited by its small sample size. For the pilot phase of the PLAY Study, we were unable to recruit a larger sample size due to the time and financial resources required to collect, process and analyse the headcam and accelerometer data, as well as to develop the feedback and to deliver it in a one-on-one setting. While qualitative research is less reliant on a large sample size and more reliant on information-rich discussions, we can only consider this data as preliminary to further guide the protocol for the PLAY Study trial implementation. Acceptability will be tested during and after the PLAY Study trial in a much larger sample. This study was conducted in a very specific, purposively selected population group and the findings are not generalizable. However, this has allowed for fine tuning of the feedback to make it contextually relevant and acceptable within this population. Social desirability may have biased the focus group discussions, however participants were aware that all data was anonymised in the transcription, and we took steps to ensure that the facilitator was relatable. We did elicit negative responses about the intervention, indicating that participants did not avoid providing negative feedback. While we did not aim to assess effectiveness in this short pilot study, we have provided data on participants’ self-efficacy, which is the primary outcome for the PLAY Study trial. The fact that responses changed before and after receiving feedback indicates that self-efficacy is likely a valid outcome measure for this trial, but does not indicate evidence for effectiveness given this was not a randomised trial and the sample size was selected for qualitative analysis only. We did not include a patient and public involvement group in the first stage of the feedback development, however we have used the findings from this pilot study to guide further development of the protocol for the PLAY Study trial. Future studies aiming to implement similar feedback methodologies should undergo pilot testing and involve the community in the early stages of development of consequent interventions.

In conclusion, we have been able to develop feedback on two infant behaviours with the potential to sensitise mothers to their infants’ behaviours, as well as to improve their self-efficacy through increasing skills, feelings of competence and reflexive functioning. The feedback was found to be both feasible and largely acceptable, however based on the learnings from this pilot study modifications to the protocol have been made to improve implementation and hopefully increase acceptability of the PLAY Study trial.. Future studies aiming should examine acceptability in a larger sample, and determine the scalability of this personalised intervention delivered in one-on-one sessions with supportive guidance. If found to be effective, these feedback methodologies could be adapted and implemented in diverse contexts in the future.

## Ethical approval

Ethical approval was granted (M210846) by the Human Ethics Research Committee of the University of the Witwatersrand, Johannesburg, South Africa on the 25/02/2022. This research was conducted in accordance with the Declaration of Helsinki.

## Data Availability

OSF: Behavioural feedback intervention pilot. https://doi.org/10.17605/OSF.IO/EC78S ^51^. This project contains the following underlying data ^51^: Transcript_Feedback_Mothers Group (Full transcript of FGD session) Feedback_Analytical dataset (Demographic data, acceptability questionnaire data and self-efficacy data) Feedback_Data dictionary (Data dictionary for each variable) Data are available under the Creative Commons Attribution 4.0 International Public License.
