# Supplementary material for: “I always wondered if my baby is able to feel my love for them” -  Development and pilot testing of two behavioural feedback strategies designed to improve maternal self-efficacy
Source: Wellcome Open Res. 2025 May 9;10:47. Originally published 2025 Feb 7. [Version 2] doi: 10.12688/wellcomeopenres.23564.2 (PMC12120436; doi:10.12688/wellcomeopenres.23564.2)
Supplement: Peer review history

# Reviewer report: Reviewer response for version 2

**DOI:** 10.21956/wellcomeopenres.26673.r123349

**Published:** 2025-05-28

**Authors:** Phillip Harniess

**Affiliations:** 1 University of Exeter, Exeter, UK

Thank you for making the edits as suggested.

Is the study design appropriate for the research question?

Yes

Is the rationale for, and objectives of, the study clearly described?

Yes

Are sufficient details of the methods provided to allow replication by others?

Yes

Are the datasets clearly presented in a useable and accessible format?

Not applicable

Reviewer Expertise:

Early intervention physical therapies for families of infants at risk of neurodevelopmental sequelae. Family centred care, qualitative research and implementation science.

I confirm that I have read this submission and believe that I have an appropriate level of expertise to confirm that it is of an acceptable scientific standard.

# Reviewer report: Reviewer response for version 2

**DOI:** 10.21956/wellcomeopenres.26673.r123350

**Published:** 2025-05-24

**Authors:** Caitlin Canfield

**Affiliations:** 1 New York University School of Medicine, New York, USA

The authors have addressed all of my prior concerns, and their findings provide a compelling first step for interventions to improve maternal self-efficacy related to infant movement and development.

Is the study design appropriate for the research question?

Partly

Is the rationale for, and objectives of, the study clearly described?

Yes

Are sufficient details of the methods provided to allow replication by others?

Yes

Are the datasets clearly presented in a useable and accessible format?

Not applicable

Reviewer Expertise:

Developmental psychology and early parenting interventions

I confirm that I have read this submission and believe that I have an appropriate level of expertise to confirm that it is of an acceptable scientific standard.

# Reviewer report: Reviewer response for version 1

**DOI:** 10.21956/wellcomeopenres.25996.r118837

**Published:** 2025-03-10

**Authors:** Phillip Harniess

**Affiliations:** 1 University of Exeter, Exeter, UK

Thank you for the opportunity to review this paper – short title, “ *I always wondered if my baby is able to feel my love*”.

 Congratulations on carefully presenting this work around an important topic that has significant sensitivities, not least the power asymmetries in the research processes themselves. Overall it is well written, although I picked up numerous typographical errors that must have escaped the spell check (e.g. Poositive, comducted breastfeeding, relevent, likely, hygeine).

 Within the introduction at the end of the first paragraph you state:

 “Socially vulnerable caregivers may find it more challenging to become attuned to infant cues or to feel confident that they can promote a stable environment due to the pressures of their daily lives.”

 Is this the hypothesis of the authors or is there a body of research to support this statement, I’m sure it is unintentional, but it reads as a jump to an assumption. Why may socially vulnerable caregivers find it difficult to become attuned to infant cues? I think later in the introduction this is addressed but not fully in the context of this paragraph here.  

 It was interesting in the methods that experts/attendees “agreed that graphical focus on daily routines, variations in routines, sleep hygeine, and peaks of high activity (potentially play) would assist with delivering the key feedback messages.” Were there any PPI members with lived experience involved in this early design process as well?

 Presumably data literacy was a relevant challenge. It was intriguing that mothers found the graphical feedback helpful – how much support was needed around the presentation and interpretation of these quite abstract graphs with the mothers. To what extent did the mothers interpret these data independently to guide behaviour change and to what extent was the message interpreted for them? This is relevant for the scalability of any such intervention where additional support is limited.

 Eight consented to participate in what appears to be a challenging context for recruitment to research, especially around the sensitivity of parenting. It was good to see that positionalities were considered in the researcher characteristics and I think it is clear how that was managed through the intervention. Was the acceptability questionnaire blinded? To what extent do you think a limitation was social desirability, especially in relation to the power dynamics?

 The results demonstrate a good balance between positive feedback and the challenges that the mothers faced during the intervention. There is a rich catalogue of relevant phenomena of interest. And I think the results and including discussion draws well on most of the queries I had in relation to the affect of the camera on the infant behaviour, the effect of using the camera on the mother’s behaviour as well as the issue of the normative construct of routines in parenting and some *interactions that may not feel natural to mothers. *Some the presentation in results is challenging for the reader, where back-back quotes are provided, these could be reduced or place in a table potentially.

 One area that I believe was overlooked however was the impact of the intervention on maternal wellbeing and stress. Was there a reason this wasn’t explored in detail, for feasibility reasons? It would be helpful to understand to what extent the intervention affected maternal stress for better or worse. Of course, we might hope/hypothesise the parental wellbeing would improve through this experience. However, there is also a potential dark logic – where unintended consequences of introducing such an intervention (with all  of its normative assumptions) might increase maternal stress / guilt in their parenting experience, especially as they seek and find it challenging to regulate their own emotion through interaction with their child.

 It was not clear how you defined the construct of acceptability except that you were looking to understand more about the feedback element of the intervention. I believe use of a framework, such as the theoretical framework of acceptability (TFA) (Sekhon et al., 2017) would be an important for a comprehensive view in this study where acceptability is so central.

 Overall, I think this is a good first step in terms of usable information to inform the intervention – there is a rich seam of data and insights to work with. There are bigger questions/challenges in terms of future implementation and scalability that it would be good for the authors to consider in their discussion further.

Is the study design appropriate for the research question?

Yes

Is the rationale for, and objectives of, the study clearly described?

Yes

Are sufficient details of the methods provided to allow replication by others?

Yes

Are the datasets clearly presented in a useable and accessible format?

Not applicable

Reviewer Expertise:

Early intervention physical therapies for families of infants at risk of neurodevelopmental sequelae. Family centred care, qualitative research and implementation science.

I confirm that I have read this submission and believe that I have an appropriate level of expertise to confirm that it is of an acceptable scientific standard, however I have significant reservations, as outlined above.

## References

[1] : Acceptability of healthcare interventions: an overview of reviews and development of a theoretical framework. *BMC Health Serv Res* .2017;17(1) : 10.1186/s12913-017-2031-8 88 10.1186/s12913-017-2031-8 28126032 PMC5267473

# Response

**Published:** 2025-04-25

**Authors:** Alessandra Prioreschi

**Affiliations:** Pediatrics, University of the Witwatersrand Johannesburg School of Clinical Medicine, Johannesburg, Gauteng, South Africa

Thank you for the opportunity to review this paper – short title, “I always wondered if my baby is able to feel my love”.

 Congratulations on carefully presenting this work around an important topic that has significant sensitivities, not least the power asymmetries in the research processes themselves. Overall it is well written, although I picked up numerous typographical errors that must have escaped the spell check (e.g. Poositive, comducted breastfeeding, relevent, likely, hygeine). *Thank you for pointing these out. We have now undertaken proofreading of the manuscript and corrected typographical errors.*   Within the introduction at the end of the first paragraph you state:

 “Socially vulnerable caregivers may find it more challenging to become attuned to infant cues or to feel confident that they can promote a stable environment due to the pressures of their daily lives.”

 Is this the hypothesis of the authors or is there a body of research to support this statement, I’m sure it is unintentional, but it reads as a jump to an assumption. Why may socially vulnerable caregivers find it difficult to become attuned to infant cues? I think later in the introduction this is addressed but not fully in the context of this paragraph here. *We have now added references to support this statement, and have expanded the statement slightly for clarification.  *   It was interesting in the methods that experts/attendees “agreed that graphical focus on daily routines, variations in routines, sleep hygiene, and peaks of high activity (potentially play) would assist with delivering the key feedback messages.” Were there any PPI members with lived experience involved in this early design process as well? *There were unfortunately no PPI members with lived experience in the first stage of the design, however the purpose of this pilot study was to gain insight from mothers in the community in order to guide further development of the feedback protocol. The findings from this pilot study were used in the development of the PLAY Study trial, which is an RCT using this feedback approach in the first year post-partum with the goal of improving maternal self-efficacy. We have worked to make this clearer in the methods, and have mentioned the limitations of not including patient and public involvement in the earlier design.*

   Presumably data literacy was a relevant challenge. It was intriguing that mothers found the graphical feedback helpful – how much support was needed around the presentation and interpretation of these quite abstract graphs with the mothers. To what extent did the mothers interpret these data independently to guide behaviour change and to what extent was the message interpreted for them? This is relevant for the scalability of any such intervention where additional support is limited. *We have added to the methods section to explain how the graphs were explained. Mothers were provided with support in interpreting the information, but we also ensured that they understood and could point out patterns in the graphs that made sense to them. This intervention was designed to be delivered in person with guided feedback to promote the strengths based messages. This does affect scalability. We have now discussed scalability in the discussion of the manuscript. Furthermore, the fidelity of the PLAY Study trial has been evaluated and is in preparation for publication. The fidelity manuscript speaks more to the scalability based on data from the fully executed randomised controlled trial which was delivered at a much larger scale over a 12 month period.*

   Eight consented to participate in what appears to be a challenging context for recruitment to research, especially around the sensitivity of parenting. It was good to see that positionalities were considered in the researcher characteristics and I think it is clear how that was managed through the intervention. Was the acceptability questionnaire blinded? To what extent do you think a limitation was social desirability, especially in relation to the power dynamics? *Yes the acceptability questionnaire was blinded, and we have now mentioned this in the methods. It is possible that social desirability somewhat biased the focus group discussions, however these sessions were run by trained research assistant who was a similar age to participants, from the same community, and also a mother of young children. While the principal investigator was present, she did not understand the languages being used in the FGDs (there are 11 official languages in South Africa!). Participants were aware that all FGD transcriptions were anonymised and that participants were given numbers by the transcriber to differentiate between speakers. We did elicit negative responses from the discussions, indicating that participants were not avoiding giving negative feedback. We have added this point to the limitations section. *   The results demonstrate a good balance between positive feedback and the challenges that the mothers faced during the intervention. There is a rich catalogue of relevant phenomena of interest. And I think the results and including discussion draws well on most of the queries I had in relation to the affect of the camera on the infant behaviour, the effect of using the camera on the mother’s behaviour as well as the issue of the normative construct of routines in parenting and some interactions that may not feel natural to mothers. Some the presentation in results is challenging for the reader, where back-back quotes are provided, these could be reduced or place in a table potentially. *Thank you for this suggestion. We have now removed a large portion of the quotes from the results text, and added them to a new Table (Table 2).*

   One area that I believe was overlooked however was the impact of the intervention on maternal wellbeing and stress. Was there a reason this wasn’t explored in detail, for feasibility reasons? It would be helpful to understand to what extent the intervention affected maternal stress for better or worse. Of course, we might hope/hypothesise the parental wellbeing would improve through this experience. However, there is also a potential dark logic – where unintended consequences of introducing such an intervention (with all  of its normative assumptions) might increase maternal stress / guilt in their parenting experience, especially as they seek and find it challenging to regulate their own emotion through interaction with their child. *This is an important point to consider. While we did not measure maternal stress in this pilot study, maternal anxiety and stress are being measured in the PLAY Study trial and we will be able to evaluate the effect of the intervention on these maternal outcomes from the trial data. Given this was a pilot study with a small sample size and no control group, designed only to test feasibility and acceptability of the feedback, we would not have been able determine any effect of the feedback. Furthermore, we specifically chose to focus on positive interactions only (strengths based approach), as a means to promote feelings of self-efficacy and show mothers how well they were doing. The hypothesis was that this would reduce negative emotional responses. We are hopeful that any negative experiences from receiving the feedback would be captured by the acceptability questionnaires and the focus group discussions, and did indeed learn about some scenarios in  the video feedback which made mothers unhappy. We have added some text on this point to the methods.*

   It was not clear how you defined the construct of acceptability except that you were looking to understand more about the feedback element of the intervention. I believe use of a framework, such as the theoretical framework of acceptability (TFA) (Sekhon et al., 2017) would be an important for a comprehensive view in this study where acceptability is so central. *Thank you for this suggestion. We have now included an interpretation of acceptability based on Sekhon’s framework. This is included in the methods, and in the discussion. We have restructured the entire discussion to focus on the results in terms of the constructs of acceptability.*

   Overall, I think this is a good first step in terms of usable information to inform the intervention – there is a rich seam of data and insights to work with. There are bigger questions/challenges in terms of future implementation and scalability that it would be good for the authors to consider in their discussion further. *We have added discussion around factors to consider for implementation of the PLAY Study trial and future interventions, as well as issues related to scalability.*

# Reviewer report: Reviewer response for version 1

**DOI:** 10.21956/wellcomeopenres.25996.r118841

**Published:** 2025-03-10

**Authors:** Caitlin Canfield

**Affiliations:** 1 New York University School of Medicine, New York, USA

This manuscript, "’I always wondered if my baby is able to feel my love for them’ -  Development and pilot testing of two behavioural feedback strategies designed to improve maternal self-efficacy," presents findings from a small pilot study of a program to provide feedback on maternal and infant movement and interaction in order to build maternal self-efficacy.

 The study presents interesting qualitative findings. However, no formal qualitative or quantitative analyses were conducted, likely due to the very small sample size (n = 7). I am also unclear as to whether this manuscript meets the criteria for a study protocol, as it reports the findings of a pilot study, rather than the methodological protocol for one.

 There were also a few other more minor concerns with the manuscript, as outlined below:

1. The authors did not adequately set up the need or importance of education and self-efficacy around movement. The methods note that guidelines around movement (e.g., 30 of moderate/vigorous activity per day) were shared with mothers during focus group discussions. However, there is no review of literature related to the importance of movement/physical activity for parents or infant development.
2. There were several typos through the manuscript. The authors should seek out additional proofreading for any manuscript revision.
3. The Discussion sometimes goes beyond the actual findings of the study. The authors should be careful about drawing broad conclusions given the extremely small sample size and lack of formal analyses to indicate differences from pre- to post-intervention.

Is the study design appropriate for the research question?

Partly

Is the rationale for, and objectives of, the study clearly described?

Yes

Are sufficient details of the methods provided to allow replication by others?

Yes

Are the datasets clearly presented in a useable and accessible format?

Not applicable

Reviewer Expertise:

Developmental psychology and early parenting interventions

I confirm that I have read this submission and believe that I have an appropriate level of expertise to state that I do not consider it to be of an acceptable scientific standard, for reasons outlined above.
